# Pathological Bases for a Robust Application of Cancer Molecular Classification

**DOI:** 10.3390/ijms16048655

**Published:** 2015-04-17

**Authors:** Salvador J. Diaz-Cano

**Keywords:** molecular pathology, cancer, neoplasm, classification, analytical genomic classification of tumors, genomic pathology, cancer cell biology

## Abstract

Any robust classification system depends on its purpose and must refer to accepted standards, its strength relying on predictive values and a careful consideration of known factors that can affect its reliability. In this context, a molecular classification of human cancer must refer to the current gold standard (histological classification) and try to improve it with key prognosticators for metastatic potential, staging and grading. Although organ-specific examples have been published based on proteomics, transcriptomics and genomics evaluations, the most popular approach uses gene expression analysis as a direct correlate of cellular differentiation, which represents the key feature of the histological classification. RNA is a labile molecule that varies significantly according with the preservation protocol, its transcription reflect the adaptation of the tumor cells to the microenvironment, it can be passed through mechanisms of intercellular transference of genetic information (exosomes), and it is exposed to epigenetic modifications. More robust classifications should be based on stable molecules, at the genetic level represented by DNA to improve reliability, and its analysis must deal with the concept of intratumoral heterogeneity, which is at the origin of tumor progression and is the byproduct of the selection process during the clonal expansion and progression of neoplasms. The simultaneous analysis of multiple DNA targets and next generation sequencing offer the best practical approach for an analytical genomic classification of tumors.

## 1. Introduction

A general definition of neoplasm like “cellular disease characterized by abnormal growth regulatory mechanisms” is descriptive and difficult to apply routinely, working definitions being required. The introduction of new markers has improved the diagnostic precision, but can potentially result in big changes in prevalence and uncertainties for particular lesions. The current WHO classifications of tumors incorporate new developments based on pathology and genetics, the leading criteria still being morphological; in this context, molecular findings complement the histological evaluation without replacing it. Additionally, any new definition should be validated against the accepted standard (specificity/sensitivity), should improve patient’s management, and should provide a biologic meaning for its application. The first requirement is typically met by the initial design, and the second condition would be expected in any successful implementation. The third criterion is harder to put into practice, but any new definition should be biologically meaningful and would incorporate core elements in tumor biology (in particular genetic and kinetic correlates) [1,2,3,4,5]. These elements need to be included in a score system. Examples include the PAX8/PPARγ fusion gene described in follicular thyroid carcinomas and adenomas or RET/PTC fusion genes reported in papillary thyroid carcinomas and Hashimoto’s thyroiditis. Molecular findings are contributing to a better understanding and classification of neoplasms [6,7,8]. The proposal, to classify neoplasms based only on the identification of one molecular abnormality, is naïve, especially considering that neoplasms require several mutations to reach the no-return point during the malignant transformation [4,5,9,10]. This approach raises fundamental issues in tumor classification and terminology that would need further considerations. Any classification is an organization in domains by hierarchical groups, according to features generalizable to the members of the groups. Classifications are important because class properties are shared among the members of a class, and because members of a class inherit the properties of their ancestors. The classification process must also be differentiated from terms like identification, discrimination, taxonomy, and ontology that cause considerable confusion among pathologists and cancer researchers. Identification is the act of placing something into its correct slot within an existing classification. Discrimination is finding features that separate members of a group according to expected variations in group behavior, the so-called prognostic factors such as grading and staging. A taxonomy is a complete listing of all the members of a classification, and an ontology is a rule-based grouping of some portion of a taxonomy.

## 2. General Features of Tumor Classification: Current Status

A classification has certain properties that distinguish it from other ways of organizing data. Any classification tries to encapsulate all knowledge related to a domain, but much of the current work in the molecular classification of tumors is identification, and discriminant analysis disguised as classification. It is impossible to create a molecular classification of tumors based solely on the separation of tumors by variations of molecular markers. Clustering by variation only identifies differences among tumors and is not sufficient to establish a classification. Classification is the process of showing that certain differences reliably distinguish the members of a group from the members of all other groups and that these differences apply to the group’s hierarchical descendants. In a modern classification, the elements of the classification serve as annotation keys and are capable of relating all data to the classification, incorporating molecular pathways that will be used as targets for new, non-toxic chemotherapeutic agents. There have been early successes with tumors sensitive to the inhibition of tyrosine kinases (gastrointestinal stromal tumors (GIST) and chronic myelogenous leukemia) with imatinib [11]. Both these tumors derive from non-endodermal/ectodermal embryonic layers, suggesting that molecular pathways (hence targets for chemotherapy) may be class-dependent.

Reliable classification systems require a careful use of internationally accepted nomenclature based on differentiation-developmental system that represents the current gold standard. The main criteria used in tumor classification are founded in tissue differentiation and developmental biology [4,9,10,12,13], which result in a system that gives several advantages: assign tumors to unique positions within the classification, avoiding the problem of multiple embryologic derivatives that can be present within one organ; provide a cell developmental stage that is usually reproduced by the differentiation process; give relevant behavioral information, such as the likely metastatic dissemination via lymphatics for epithelial neoplasms and by hematogenous spread of mesenchymal neoplasms; correlate closely with modern molecular analysis of tumors (sarcomas and lymphomas are frequently characterized by simple fusion genes, carcinomas and melanomas cannot be marked by a single genetic abnormality, and primitive blastomas share similar markers regardless of the organ of origin). For these reasons, the classification nomenclature used internationally is based on this terminology. However, current tumor classifications are site or organ system specific, often based on medical disciplines rather than biologic principles, appear redundant when subclassifications are merged and have problems analyzing heterogeneous biological data. Several fundamental issues in tumor classification exemplify the growing rift between morphologic and molecular approaches to tumor classification [9,10,14,15]: (1) intratumor heterogeneity and morpho-functional changes associated with tumor progression; (2) the distinction between loss of differentiation during progression and primary lack of differentiation; (3) the molecular properties shared by morphologically disparate tumors with or without a common developmental lineage; (4) the grouping of tumors based on shared cellular functions; (5) the separation of epithelial and non-epithelial tumors; (6) the distinction between germ cell tumors and pluripotent tumors of non-germ cell origin; and (7) the problem of re-classifying morphologically identical but clinically distinct subsets of tumors. If a tumor lacks a morphologic or molecular feature at one point in its development and gains it at a later point, the element cannot determine a new class of tumor. If a tumor has a good prognosis at one point (e.g., before it has metastasized) and a bad prognosis later (e.g., after it has metastasized), then prognostic features associated with metastasis cannot be used to determine a new class of tumor. 

The differentiation-developmental classification of neoplasms makes basic assumptions, including: morphologic and molecular features of tumors will both fall sensibly into classes determined by tumor cell lineage; pathways with molecular alterations producing a tumor phenotype will tend to operate in all tumors of a developmental class; and morphologic properties associated with the altered pathway will be visible for all class members. It would therefore be sensible to keep the advantages of morphological classifications, complementing it with biological concepts and measurable features provided by molecular analysis, in particular those coming from genomic evaluations (multigene analysis highlights cooperative genetic interactions) [9,10]. Our understanding of embryologic lineage has changed very little over the past half century, and a classification based on differentiation and developmental biology permits tumors to be assigned to well-defined classes. Somatic DNA has lineage-specific epigenetic modifications that occur throughout development [16,17,18,19,20], opening the avenue for measurable and definable patterns of epigenetic modifications (e.g., methylation) as key informative features for the developmental lineage of tumors. Kho and coworkers [13] have developed a method that projects gene expression profiles of tumors onto a mouse developmental sequence. Human medulloblastoma most closely matched the gene expression profile of postnatal day five mouse cerebella. Although this study examined only a few tumors, it described a method that allows any human tumor to be matched against a library of gene expression profiles collected from normal tissues at different stages of development.

Because classifications are hypotheses about the fundamental nature of a knowledge domain, the foundational assumptions of any classification must be continually evaluated and challenged. In this scenario, the role of pathologists is essential as the best link to accomplish a robust, reliable and biologically founded classification of neoplasms. The pathologist prepares, describes and diagnoses the tissue samples; organizes and integrates the available information (demographic, clinical, and ancillary techniques); and identifies enriched tissue samples for more reliable molecular results by microdissection techniques. In all cases, the clinicopathologic annotations used by the molecular biologist are generated in whole or in part by surgical pathologists. It has been noted that, “the pathologist’s understanding of anatomic, physiologic, biochemical, immune, and other underlying factors that drive mechanisms of tissue responses to noxious agents turns a bewildering array of gene expression data into focused research programs” [21].

## 3. Key Cellular and Molecular Processes to Incorporate in a Robust Classification

**Figure 1:**
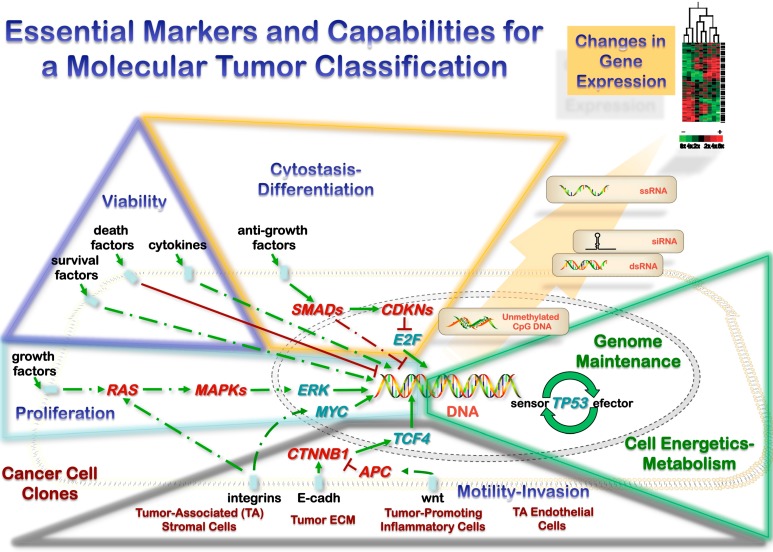
Malignant tumors are heterogeneous biological structures comprising cancer cell clones along with tumor-associated components necessary for maintaining the autonomous growth (stromal cells, extracellular matrix, inflammatory cells and endothelial cells). This heterotypic biology is self-maintained by acquired capabilities that promote both endless growth (proliferation, expanded cell survival or viability, resistance to cytostasis, and motility/invasion) and progression (evasion of genome maintenance, and appropriate cell energetics-metabolism). Cooperative genetic alterations in these pathways are common in cancer at different levels (DNA sequence, chemical modifications of DNA and gene expression), and their combinations can serve as elements of a molecular classification. However, as these combinations are not always pathognomonic and there is an additional complexity of gene expression (intercellular RNA transfer in heterotopic biology, RNA degradation), multitargeted simultaneous DNA analysis would be the most promising way to improve our current pathological neoplasm classification.

Biologically, neoplasms develop through acquisition of capabilities that involve tumor cell aspects and microenvironment interactions [9,22,23]. The unrestricted growth observed in neoplasms is generally due to a stepwise accumulation of cooperative genetic alterations in oncogenes and tumor-suppressor genes, the number being more important than the order of changes; [24] the evidence available suggest that 5–7 genetic alterations are required for clinically detectable tumors, correlating with morphological progression in some locations. These capabilities are not equally relevant at different stages during tumorigenesis, as highlighted by careful morphological evaluations. The markers should be selected considering the ability to test and the marker role during tumor initiation and promotion. Tumor promotion markers would be more relevantly assessed during progression, which needs to be defined on clear clinical and morphological grounds. The main tumorigenesis molecular pathways must be evaluated according to the acquired capabilities: self-maintained replication (cell cycle dysregulation), extended cell survival (cell cycle arrest, apoptosis dysregulation, and replicative lifespan), genetic instability (chromosomal and microsatellite), changes in chromatin, transcription and epigenetics, mobilization of cellular resources, and modified microenvironment interactions (tumor cells, stromal cells, extracellular, endothelium) [9]. All these aspects must finally be integrated into the mechanisms of tumor initiation (including clonality) and progression. The knowledge of these pathways in each acquired capability is also essential to plan any sensible molecular evaluation of neoplasms (Figure 1): It will allow a marker selection based on biological features and it will allow a precise range of surrogate/secondary markers to validate the results. In addition, some pathways are mutually exclusive (*i.e.*, RAS and B-RAF mutations or EGFR and RAS analyses) and have to be evaluated simultaneously.

Molecular markers and pathways should be selected based on biological criteria comprising key features of tumorigenesis: heterotypic biology including microenvironment interactions and their role in mutagenesis (as key aspects of activation/suppression of gene function) and metastatic potential, along with the gene expression deregulations and their role in differentiation. These elements then need to be integrated with a sensible molecular test requirement and score system for practical implementation.

### 3.1. Heterotypic Tumor Biology: Tumor Cells-Microenvironment Interactions

Normal cells survive and grow within defined environmental niches and are subjected to microenvironmental control. Outside of their particular niche, the tissue environment is hostile to normal cells. Since they lack necessary cell autonomous survival signals, normal cells will not survive an inappropriate microenvironment [25]. Detachment-induced cell death (anoikis) has been proposed as the mechanism preventing normal cells from leaving their original environment and seeding at inappropriate locations [9]. In order to evade local tissue control and avoid anoikis during tumor development and progression, malignant cells start interacting with the surrounding ECM [26]. A bidirectional relationship is initiated by tumor cells and its surrounding stroma as a first step to invasive growth on metastatic spreading. Stromal changes sustaining tumor progression include modifications of the ECM composition, activation of fibroblasts, myoepithelial cells, and the recruitment of pericytes or smooth muscle cells and immune and inflammatory cells [27].

Human tumors arise from single cells that have accumulated the necessary number and types of heritable alterations. Each such cell leads to dysregulated growth and eventually the formation of a tumor. Despite their monoclonal origin, at the time of diagnosis most tumors show a striking amount of intratumor heterogeneity in all measurable phenotypes; the evolutionary dynamics of heterogeneity arising from exponential expansion of a tumor cell population, in which heritable alterations confer random fitness changes to cells [28]. Classical multistage modeling of tumorigenesis evolves through the processes of local proliferative lesions (tumor initiation and promotion or selection), and acquisition of invasion-metastatic potential (tumor progression). Broadly speaking, tumor development consists of the selective (clonal) expansion of altered cells to form focal lesions [3,29]. Within this definition, the process of promotion is mainly a quantitative phenomenon (many cells arising from a single cell), while no qualitative changes are necessarily implied. However, these latter properties are lost during tumor progression, which is typically characterized by increasing levels of tumor cell heterogeneity resulting in dominant qualitative changes [5,30], generating distinct cellular subclones with different phenotypes. Such a background represents the landscape for the full deployment of tumor progression.

These two distinctive processes, the mainly *quantitative* process of tumor promotion and the intrinsically *qualitative* process of tumor progression, are driven by two distinct microenvironments: the tissue and the tumor microenvironments [31,32,33]. The tissue microenvironment specifically refers to the local environment surrounding altered cells during their selective clonal expansion to form focal proliferative lesions. Conversely, the tumor microenvironment describes the unique biological milieu that emerges inside focal proliferative lesions as a consequence of their altered growth pattern [31,32,33]. Such new biological niche is characterized by a tissue architecture, which is not developmentally programmed and is bound to pose significant challenges for cell survival, due to altered/inadequate supply of oxygen and nutrients. This in turn can lead to biochemical and metabolic alterations that can profoundly impact on the fate of the cell populations inside focal lesions [34].

Given that altered cells can be selected in a tissue microenvironment which is otherwise growth-inhibitory to surrounding counterparts, a relevant question pertains to the biochemical and molecular basis of such phenotypic resistance. Blagosklonny has proposed the existence of two broad types of resistance [35]: (I) Non-oncogenic resistance relates to changes in drug metabolism and/or uptake, such that the rarely altered cell can withstand toxicity compared to the rest of the population in that tissue. Such phenotypic resistance would still translate into the clonal growth of that rare cell, but no increased risk of neoplastic disease would be implied [35]; (II) The oncogenic resistance is linked to the inability of the cell to sense or repair DNA damage and/or to activate effector mechanisms leading to cell cycle arrest and/or cell death. As a result, the affected cell is susceptible to acquire a “mutator phenotype”, *i.e.*, the tendency to undergo a cascade of further mutations [9,36,37,38].

The mutator phenotype has been linked with a defect in mismatch repair (MMR) genes so that a cascade of mutations occurs in cancer-related genes. To justify the onset of a mutator phenotype in “sporadic cancers” (which are in fact the vast majority) we have to revisit some theories of carcinogenesis and their evidence base [1,39]. In sporadic cancers, the origin of the mutator phenotype has been attributed to chance, or to mutagens that selectively affect specific genes similar to MMR genes, or to a combination of the two. However, MMR is apparently mutated only in a minority of cases: For example, colon cancers characterized by the presence of microsatellites (MIN) are a small minority compared to cancers characterized by chromosome instability (CIN), whose onset has not yet been attributed to the failure of any particular gene repair such as MMR [37,38]. To explain the most common type of lesions that are found in nonhereditary cancers, chromosome aberrations, and CIN, we have to explain how the mutator phenotype originates. In addition, a fundamental concept emerging recently is that mutations or instability are irrelevant if there is not a *microenvironmental change* that selects the cells carrying such mutations.

Cell replication is the primary source of cellular stress. On one hand, continuous proliferation results in telomere attrition and reduced stability of chromosome ends, which activate the cycle of chromosomal fusion-bridge-breakage and a higher incidence of translocations such as expression of chromosomal instability (CIN). On the other hand, nucleotide mismatches are introduced by DNA polymerase and will accumulate in DNA regions with repetitive sequences, such as microsatellites; this is the primary reason for microsatellite instability (MSI), a finding more frequently detected in tissues with higher proliferation. CIN and MSI have been described as two alternative pathways to cancer [9,38]. CIN is defined as the ability of a cell to gain and lose chromosomes and is a feature of many types of cancer. Conversely, microsatellite instability is related to a defect in the DNA mismatch repair machinery (MSI cancers).

The net result of CIN is the deregulation of chromosome number (aneuploidy) and an enhanced rate of loss of heterozygosity, which is an important mechanism of inactivation of tumor suppressor genes. Cytogenetic studies of bladder, lung and colon tumors have shown that karyotype complexity, cell ploidy, and the number of structural changes found were closely associated with tumor grade and stage. It has been suggested that different environmental carcinogens can induce distinct forms of genetic instability [40]. The available data demonstrate that exposure to particular carcinogens can indeed select for tumor cells with distinct types of genetic instability and *vice versa*. These data offer potential clues to one of the remaining unsolved problems in cancer research, the relationship between environmental factors and the genetic abnormalities that affect the tumorigenesis.

Chronic inflammation promotes tumor onset and development through nonimmune and immune mechanisms. The nonimmune mechanisms include the following: (I) the production of reactive oxygen species (ROS) such as peroxynitrites, which cause DNA mutations that contribute to genetic instability and the proliferation of malignant cells [41]; (II) the production of proangiogenic factors such as vascular endothelial growth factor (VEGF), which promote tumor neovascularization [42]; and (III) the production of matrix metalloproteases, which facilitate invasion and metastasis [43].

### 3.2. Microenvironment and Metastasis

Cancer is a systemic disease: malignant tumors shed large numbers of cells into the blood and lymph vessels, some of them developing in distant sites into metastases. Moreover, distant metastasis is responsible for the majority of cancer-related deaths, and, therefore, understanding the underlying biological mechanisms of it is of primary importance. The invasion/metastasis capability is closely related with cell motility and requires the cytoskeleton as a critical component, which is also essential during mitoses. As malignancy criteria are mainly related to the phenotype of actively proliferating cells, it not surprising that metastatic deposits genetically match well-differentiated areas of primary neoplasms, and that invasive areas (periphery of solid organ neoplasms and deep compartment of luminal organ tumors) show lower cellular turnover and higher incidence of genetic abnormalities [38,39,44,45,46]. These factors need attention when planning the evaluation of intratumoral heterogeneity and would include: detailed specification of sampling (intratumoral location, number of samples), combined evaluation of kinetic and genetic features to assess selective process, analysis of pathways at several steps to avoid confounding factors (redundancy and pleiotropism) [5,9,47]. These biological foundations will enable a better therapeutic design, using the heterogeneity to improve patient’s management. 

According to a traditional model of tumor development, tissue constraints constitute a significant evolutionary bottleneck in cancer evolution; thus, the acquisition of metastatic ability is considered to be the final step in tumor development, contingent on the acquisition of all of the other hallmarks of cancer [9,22,23]. This model implies that metastatic tumors should be genetically similar to the bulk of primary tumor cells.

Many studies that compared the genetic composition of primary tumors and secondary metastatic sites have found very close clonal relationships between the two in the majority of cases [48,49,50]. Similarly, analysis of gene expression profiles revealed very similar patterns between primary tumors and metastatic sites, a scenario highly unlikely for genetically divergent clones [51,52,53]. Another prediction from the linear model of tumor progression is that different metastases should display close clonal relationships among each other. Indeed, this prediction is supported by a recent study that compared the genetic composition of anatomically distinct metastatic lesions in 29 prostate cancer patients using SNP arrays and CGH [54]. In all cases, different metastatic lesions from the same patients demonstrated close clonal relationships, signifying monoclonal origin [54]. This demonstration of monoclonality of metastatic cancers is especially impressive given that primary prostate cancers are frequently multifocal [55], and show substantial intra-tumor genetic heterogeneity [55,56].

While the evidence of the close genetic relationship between primary and metastatic tumors is compelling, some cases display dramatic divergence, challenging the model where acquisition of metastasis is considered to be the last step of tumor progression. Radically different patterns of allelic losses, indicative of a high degree of genetic divergence, have been reported in primary tumors and lymph node metastases in prostate cancers [49], and between primary tumors and asynchronous metastases in breast cancers [50]. Highly divergent clonal evolution was also evident in a subset of cases in CGH studies of primary tumors *versus* lymph node metastases in breast cancers and of primary tumors *versus* metastatic tumors in renal cell carcinomas [48]. A recent report, comparing sequences of primary tumors and metastases in lobular breast cancers, revealed multiple mutations present only in metastases and several other mutations with increased frequency in metastatic sites [57]. Some of these genetic changes result in a higher incidence of apoptosis of tumor cells of dormant metastases (more than threefold higher) [58]. These data show that metastases remain dormant when tumor cell proliferation is balanced by an equivalent rate of cell death and suggest that angiogenesis inhibitors control metastatic growth by indirectly increasing apoptosis in tumor cells.

### 3.3. Gene Expression: Transfer of Genetic Material and Sequence-Independent Modifications

Gene expression analysis is becoming a useful tool for a better definition of neoplasms at diagnostic, prognostic and predictive levels. The identification of predictive markers of these features will help classifying neoplasms and stratifying patients for better management. However, the nature and biological meaning of these gene expression markers is not always clear: origin of the tested RNA, mechanism of RNA transference, utility for subclassification of neoplastic lesions. Gene expression analysis is becoming a useful tool for a better definition of neoplasms at diagnostic, prognostic and predictive levels [59,60,61,62,63]. The gene expression signature also reflects the *metastatic potential* in both cell lines and tumor samples [64,65,66,67,68]. First, the expression profile of low metastatic subline is distinct from that of the high metastatic subline. Metastases of the low metastatic subline closely resembled the pattern of the low metastatic primary tumor and did not “switch over” to the high metastatic gene expression profile. Thus, heterogeneity exists in this cell line/tumor, but may not be apparent as the signature reflects the majority of the tumor cells. The low metastatic subline is capable of spawning metastases, albeit at a lower rate, and its gene expression profile is therefore of significant interest. Second, the gene expression profile of the sublines as *in vitro* cultures is very distinct from the same sublines as primary tumors. While the primary tumors are a mixture of tumor cells and heterogeneous host cells (stromal cells, endothelial cells, among others), the contribution of these cells to the observed gene expression profiles should be minimal. Thus, the differences are thought to reflect changes in the tumor cells in response to the *in vivo* microenvironment. Third, comparable numbers of genes were “turned on” and “turned off” in the more highly metastatic tissues. We think about metastasis as the acquisition of traits, but the data remind us to pay equal attention to the loss of growth and differentiation-controlling genes. Many of the differentially expressed genes fall into the category of “usual suspects” for metastasis.

Cancer cells communicate with the environment through delivery of surface proteins, release of soluble factors (growth factors and cytokines), and sophisticated nano vesicles (exosomes) for the establishment of invasive tumor growth. A central question in molecular studies is to determine the *cellular origin of the target mRNA*. Two biological aspects are essential for this process: how the tumor mRNA gets there and the functionality of this mRNA. The answers to these questions are most relevant in locations where the tumor cells are scanty and the expression profile support higher tumor cell load [69]. *Membrane vesicles* derived from both tumor and host cells have recently been recognized as new candidates for critical roles in the promotion of tumor growth and metastasis [70]. The transfer of membrane components between donor and acceptor cells has been described “trogocytosis” (from Greek “trogo”, meaning “gnaw” or “bite”). Two forms of membrane transfer (trogocytosis) have been described: via nanotubes or membrane vesicles [71]. The biogenesis of membrane vesicles fundamentally distinguishes exosomes from shedding microvesicles and apoptotic blebs. The biologic heterogeneity of intraepithelial and invasive malignancies is well known at the morphologic, kinetic and genetic levels, an issue that has not been addressed in this paratumoral gene expression analysis and should warrant future studies. The gene expression markers should be evaluated in the appropriate biological context. Gene expression profiles are determined by a gene regulatory network comprising regulatory core of genes represented most prominently by transcription factors and miRNAs, which influence the expression of other genes, and a periphery of effector genes that are regulated but not regulating [72,73]. Most studies do not differentiate between these two essential groups, which can also be useful in selecting surrogate markers for a given condition [9,72]. There is a general concept to keep in mind during the analysis of gene expression: the amount of information is overwhelming, and the number of variables included in the studies significantly outnumbers the cases. In this scenario, the significant variables can be the result of a statistical selection rather than the expression of a biologically important process for a particular neoplasm. Relevant variables frequently include genes of the general metabolic activation associated with the neoplastic transformation [9,23], rather than tissue- or differentiation-specific gene variables.

*Epigenetic* dysregulation is central to cancer development and progression [74]. The best-known epigenetic marker is DNA methylation. This dysregulation includes hypomethylation leading to oncogene activation and chromosomal instability, hypermethylation and tumor suppressor gene silencing, and chromatin modification acting directly, and cooperatively with methylation changes, to modify gene expression [75]. The initial finding of global hypomethylation of DNA in human tumors was soon followed by the identification of hypermethylated tumor-suppressor genes, and then, more recently, the discovery of inactivation of microRNA (miRNA) genes by DNA methylation.

### 3.4. Molecular Test Scoring System for Practical Implementation

**Figure 2:**
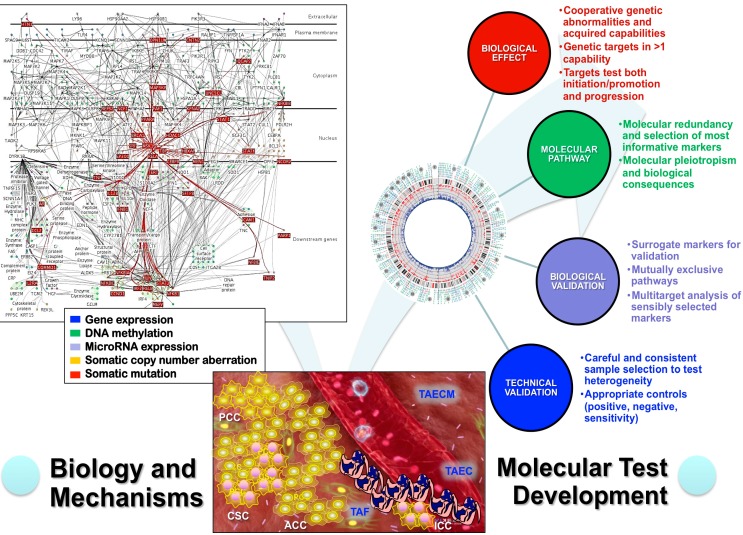
Multitarget genomic analysis of malignancies must consider the heterotypic nature of neoplasms of both cancer cells (cancer stem cells CSC, proliferating cancer cells PCC, arrested cancer cells ACC, and invasive cancer cells ICC) and tumor-associated (TA) components (fibroblasts TAF, endothelial cells TAEC, and extracellular matrix TAECM). Any comprehensive study need to address biological aspects and mechanisms of tumor genetic alterations to explain the natural history of a given neoplasm, provide essential prognostic information and predict response to therapy. The genetic alterations should be characterized at the cellular level (extracellular, plasma membrane, cytoplasm, nucleus or downstream genes) and molecular level (gene expression, methylation, microRNA, copy number aberrations and mutations). Any tests developed for this evaluation must follow a careful assessment of biological effects, pathways involved, biological validation and technical validation. In practice, the most promising approach fulfilling these criteria is the next generation sequencing.

The application of these biologic concepts in oncologic pathology leads to consider the molecular testing requirements (Molecular Test Score System) for a reliable implementation, which cover biological effects (1–3), molecular pathway (4, 5), biological validation (6–8), and technical validation (9, 10) (Figure 2) [9].

Currently, the most efficient way to achieve this goal, is by genomic approach and multi-target analysis like that provided by next generation sequencing. This multi-target analysis also complies with the multistep process widely accepted during tumorigenesis and is further supported by the need for multiple cooperative mutations in neoplasms [3,4,5,9,10,46,76]. Although organ-specific examples have been published based on proteomics, transcriptomics and genomics evaluations, the most common approach uses gene expression analysis as a direct correlate of cellular differentiation, which represents the key feature of the histological classification. RNA is a labile molecule that varies significantly according with the preservation protocol, its transcription reflect the adaptation of the tumor cells to the microenvironment, it can be passed through mechanisms of intercellular transference of genetic information (exosomes), and it is exposed to epigenetic modifications. More robust classifications should be based on stable molecules, at the genetic level represented by DNA to improve reliability, and its analysis must deal with the concept of intratumoral heterogeneity, which is at the origin of tumor progression and is the byproduct of the selection process during the clonal expansion and progression of neoplasms.

## 4. Analytical Genomic Classification of Tumors (AGCT)

An analytical genomic classification of tumors (AGCT) must be based on multiple informative features covering general characteristics of hierarchical class and instances, epidemiologic features from the pathways highlighted by inherited cancer syndromes, and the etiopathogenic features from experimental pathology (Table 1).

**Table 1:** Proposed requirements for independent classes in AGCT (Analytical Genomic Classification of Tumors).

General Features	Specific Features
**A classification is a hierarchical grouping**	Each group is defined by the greatest number of informative features that can apply to every instance of the class.
	Nomenclature should refer to differentiation/developmental terms internationally accepted.
	Every instance of the knowledge domain must fit the classification.
	Every instance and class must have exactly one slot in the classification.
	Instances of one class cannot migrate to a different class but must remain in the same class or a subclass of the same class.
	Instances and classes are separable from other instances and classes by informative features.
	All new findings of subpopulations of tumors can be considered candidate function to characterize a class and distinguish the class from other classes.
	Subclasses inherit the properties (shared informative features) of their ancestor classes.
**Familial syndromes and epidemiologic features**	Prevalence of the disease should be significantly higher in those carrying the genetic abnormalities (if familial model exists).
**Animal models of the genetic abnormality–Etiopathogenic features**	Marker gene should be more commonly abnormally expressed in animals with the disease than in controls without the disease when all risk factors are held constant.
	Incidence of the disease should be significantly higher in those animals with the abnormal gene than in those not exposed.
	A spectrum of preinvasive changes should follow the expression of the abnormal gene along a logical biologic gradient from mild to severe in the grading during neoplastic transformation (in particular for epithelial malignancies).
	Elimination or modification of the putative gene or of the vector carrying it should decrease the incidence of the disease.

A multi-target genome-wide approach warrants the simultaneous evaluation of several pathways controlling the acquired capabilities that characterize neoplasms and cancers. It needs a detailed computational bioinformatics to distinguish the driver from passenger genetic alterations, along with a quantitative analysis to assess the proportion of mutated tumor cells and copy number variation. The aim of the study should be the evaluation of affected genes and the genetic mechanisms involved in their activation/inactivation. Once candidate molecules (*i.e.*, genes, proteins, and other macromolecules or patterns of these molecules) are found to associate with a particular tumor variant, the pathologist gets a second chance to determine if a morphologic pattern correlates with the molecular property. Examples of both point mutations and translocations have been reported. Most GISTs have a *KIT* mutation that results in Kit protein overexpression [11,77]. Some GIST tumors lacking *KIT* mutations have a mutation in the platelet-derived growth-factor receptor alpha gene [78]. Sakurai and coworkers have examined GIST tumors that stain negatively for CD117, a marker for Kit protein overexpression. Many of these tumors have mutations in platelet-derived growth-factor receptor alpha gene and a distinctive histo-morphology characterized by myxoid epithelioid tumor cells and tumor infiltration by mast cells [79]. This newly recognized subtype of GIST involved the morphologic re-examination of the tumors following a molecular discovery. Secretory carcinoma of the breast is an uncommon variant of breast cancer that occurs most frequently in young women. It is characterized by the ETV6-NTRK3 fusion gene [80,81]. The search and discovery of this molecular marker was accomplished through asynchronous contributions from three biomedical realms: (1) pathologists, who defined the morphologic subset of breast carcinoma known as secretory carcinoma of breast; (2) oncologists who validated the clinically distinct features of the tumor; and (3) molecular biologists who discovered the translocation that characterized the tumor. This fusion gene is not unique for secretory carcinomas of the breast it has also been identified in radiation-induced carcinomas [82,83,84].

However, the molecular component should resolve several seemingly intractable problems. As the number of molecular pathways controlling cell-specific functions altered during the neoplastic transformation is limited, pathways will be shared by different classes of neoplasms and are not type-specific. Mutations in genes of the chromatin remodeling pathway have been described in various types of neoplasms such as malignant rhabdoid tumors [85], atypical teratoid/rhabdoid tumors of the brain [86,87,88] and hypercalcemic type of ovarian small cell carcinomas [6,7,8]; these mutations target closely related genes SMARCA and SMARCB that regulate this pathway, which is not particular of these neoplasms: SMARCB1 homozygous deletions have been found in epithelioid sarcoma and a subset of myoepithelial carcinomas [87]. Cytogenetic abnormalities and gene alterations in tumors co-occur with other anomalies, and the complex state of molecular abnormalities in tumors make it very difficult to assign classes of tumor to a single type of genetic abnormality. In addition, these alterations could represent the cause or the by-product of the genetic instability associated with the neoplastic progression, both driver and carrier mutations coexisting in the heterogeneous built of neoplasms. In this context, it may be impossible to reach scientific consensus on complex sets of molecular signatures that define groups of tumors. As an example, balanced translocations play biologic roles in several tumors [12]. Although certain translocations are characteristic of individual tumors, it has proven difficult to generalize that translocations occur in any particular class of tumors. Certainly, characteristic tumor translocations occur more commonly in mesenchymal tumors [12], but such translocations have also been observed in thyroid carcinomas [89,90], prostate adenocarcinoma and precursors [91,92], secretory carcinoma of breast [81], and in midline (lung) carcinoma of children and young adults [93]. Some translocations are also shared by carcinomas and sarcomas, such as the balanced and unbalanced chromosome X; 17 translocations in both Xp11.2 renal cell carcinoma and alveolar soft part sarcoma, supporting a preference but not a necessity for the translocation to be balanced in the carcinoma and unbalanced in the sarcoma [94,95,96]. The notable exception, wherein a class of tumors is characterized by a set of translocations, is the Ewing’s tumor family of tumors [97,98,99]. Translocations are tissue non-specific, occurring at a frequency related to the overall number of cytogenetic abnormalities found in tumors [100]. It can be noted that despite numerous projects aimed at classifying tumors with gene expression profiles, no comprehensive classification based on this technology has emerged.

Much of what passes for neoplasm “classification” in the bioinformatics literature is actually the algorithmic ranking of expressed genes that can discriminate one tumor variant from another [101,102,103,104,105], but without precise pathogenetic or etiologic considerations for those changes [14]. Some classifications can be challenged more easily than others. A classification built on a set of continually changing parameters is constantly evolving and challenging to evaluate. This is certainly true of a molecular classification, because our knowledge of the field changes almost daily. A few years ago, it was safe to say that all recurrent balanced translocations were a phenomenon of mesenchymal tumors. New findings of recurrent balanced translocations in non-mesenchymal tumors have nullified this class assertion [100]. Morphologists once classified clear cell sarcoma as a type of malignant melanoma, based on finding melanosomes within tumor cells. Recent molecular classification of these tumors clearly distinguishes them from cutaneous melanoma. Clear cell sarcomas have characteristic EWS-AFT1 fusion transcript not found in cutaneous melanomas [106,107]. In addition, BRAF mutations, commonly found in cutaneous melanomas, are absent from clear cell sarcomas [108,109]. The rapid accumulation of new knowledge about the molecular characteristics of tumors can quickly change classifications built on morphology or molecular biology. Pathologists seem to be putting this tumor back into the mesenchymal class of neoplasms [110].

## 5. Conclusions

A scientifically sound classification of neoplasms will serve as a guide to prognostic stratification of patients and to selecting a new generation of cancer medications targeted to molecular pathways specific for particular classes of tumors. Without a robust scientific foundation to the tumor classification, biological measurements on individual tumor samples cannot be generalized to other tumors, and constitutive properties common to a class of tumors cannot be distinguished from uninformative data collected from complex and chaotic biological systems (Figure 1). Morphology, even in the post-genomic era, has enormous value in the realm of discovery of informative features that eventually would be pathogenic and etiologic if possible. Careful morphologic examination has discovered previously unrecognized features that are diagnostic for new tumors or new clinical variants of known tumors that have characteristic molecular profiles. For tumor classification, lineage, morphologic, and multi-target molecular informative features must fulfill minimal criteria for the class assignment, generation of testable hypotheses and verification of etiopathogenic features (Table 1). These elements must also consider the molecular test requirements (Molecular Test Score System) for a reliable implementation, which cover biological effects, molecular pathways, biological validation, and technical validation (Figure 2).
